# Characteristics of the ideal clinical pharmacy residency candidate: A survey of residency program directors and preceptors in Saudi Arabia

**DOI:** 10.1016/j.jsps.2021.12.011

**Published:** 2021-12-31

**Authors:** Abdullah A. Alahmed, Omar S. Alkhezi, Osamah M. Alfayez, Mohammed Aseeri, Ahmad J. Mahrous, Abdulaziz M. Alhossan, John Fanikos, Abdullah Alalwan

**Affiliations:** a Department of Pharmacy Practice, College of Pharmacy, Qassim University, Qassim, Saudi Arabia; b Department of Pharmacy Services, Brigham and Women’s Hospital, Boston, MA, United States; c Department of Pharmacy Practice, College of Pharmacy-Unayzah, Qassim University, Qassim, Saudi Arabia; d Pharmaceutical Care Services, King Abdul Aziz Medical City, Jeddah, Saudi Arabia; e College of Medicine, King Saud bin Abdul Aziz University for Health Sciences, Jeddah, Saudi Arabia; f Department of Clinical Pharmacy, Umm Al-Qura University, Mecca, Saudi Arabia; g Department of Clinical Pharmacy, King Saud University, Riyadh, Saudi Arabia

**Keywords:** Pharmacy, Residency, Saudi Arabia, Candidate, Preceptor

## Abstract

**Objective:**

Residency positions are highly competitive. Pharmacy students who are familiar with the ideal qualities of residency candidates and the expectations of residency programs may be more likely to obtain one of these coveted positions. This study identifies the characteristics that residency program directors (RPDs) and preceptors use to define an ideal residency candidate.

**Methods:**

This is a cross-sectional, descriptive study that surveyed pharmacy RPDs and preceptors across the Kingdom of Saudi Arabia. The questionnaires are comprised of two sections: demographic information and characteristics of the residency candidates. Over a five-month period (May 1, 2020 – September 30, 2020), the survey was sent electronically to the participants.

**Results:**

Of the 78 surveys returned, 68 surveys were included (RPDs: 36, Preceptors: 32) and 12 surveys (15.17%) were excluded due to incompleteness. Number of RPDs responded to the survey represents (65%) of the total RPDs in Saudi Arabia. The mean response scores from the results of the Likert scale [strongly agree (1) - strongly disagree (5)] — suggest that a candidate’s performance during the interview (mean score = 1.5), their professional appearance (1.5), an alignment between a candidate’s interests and the program focus (1.6), and previous hospital experience (1.8) mattered most. While being from the same region (3.4), having an advanced degree (2.8) and the cumulative Grade Point Average (2.7) mattered the least. We find that previous hospital experience (29%), familiarity with the program (16%), research experience (15%), Saudi Commission for Health Specialists aggregate score (10%), and letters of recommendation (4%) are considered the top five factors.

**Conclusion:**

Residency candidates should focus on training in clinical settings. Offering mock interviews and Saudi Pharmacist Licensure Examination practice tests and involving pharmacy students in clinical research may increase their chance in securing a residency position.

## 1 Introduction

The number of applicants who apply to pharmacy residency programs in Saudi Arabia grow every year. An increasing number of applicants are not receiving nominations or getting matched to a residency program. Obtaining and securing a residency position is highly competitive. There are many reasons for this. First, there are a limited number of residency positions available within Saudi hospitals — with only a few new positions added each year (The Saudi Commission for Health Specialties, 2021, The Saudi Commission for Health Specialties, 2021). Based on the handful new positions compared to the vast number of applicants, this makes it unlikely that an applicant secures a position. Second, in the last fifteen years, the number of students attending pharmacy schools has increased dramatically. In 2000, there was only one pharmacy college in Saudi Arabia. In 2020, there are 28 pharmacy colleges across the country; eight colleges are private while the rest are public (“The Saudi Ministry of Education,” 3AD). Third, in many hospitals in Saudi Arabia, to work as a clinical pharmacist, residency experience or equivalent training is required. This encourages students to apply to residency programs (Badreldin et al., 2020).

Pharmacy students who possess the ideal qualities of residency candidates and understand the expectations of residency programs are more likely to obtain one of these coveted positions. The earlier students become aware of the qualities they must demonstrate and the expectations these programs have, the easier it is for them to obtain the necessary experience and skillset needed to succeed in a residency program. Faculty and hospital preceptors should also be aware of the factors considered when selecting pharmacy residents. That way, they are well-positioned to guide prospective residents and offer them opportunities to develop the proper skills before graduating.

The existing process for applying to pharmacy residency programs in Saudi Arabia is conducted through the Saudi Commission for Health Specialists (SCFHS). Residency applicants are nominated, interviewed, and then the program ranks candidates. SCHFS nominates potential candidates based on their aggregate scores. These aggregate scores include: the Saudi Pharmacist Licensure Examination (SPLE) score, Grade Point Average (GPA), and student portfolios (“The Saudi Commission for Health Specialties,” 5AD). SPLE comprises 50% of the aggregate score, while the GPA and portfolios are worth 30% and 20%, respectively. For the 2020–2021 calendar year, the portfolio consists of the following: research activity, volunteer experience, employer sponsorship, clinical experience, dean’s list awards, a stated interest in the program, and whether a student holds a postgraduate degree. Once the applicants are nominated by SCFHS, they are invited to interview with the residency programs. The residency programs also ask for supplemental materials — such as a curriculum vitae (CV), a letter of intent, and letters of recommendation. Although the factors taken into account are publicly known, how pharmacy residency candidates are ranked by hospitals remains unclear.

To date, no studies exist that identify the characteristics of an ideal clinical pharmacy residency candidate in Saudi Arabia. The same cannot be said about residency candidates applying to the United States (US) programs. Indeed, many studies in the US have assessed the qualities that residency candidates must exemplify (Gohlke et al., 2014, Pick et al., 2013). One study found that familiarity with an applicant’s pharmacy college, possessing a preexisting relationship with that specific hospital, compelling letters of recommendation, and excellent letters of intent — are essential to becoming a good residency candidate (Gohlke et al., 2014). When selecting a candidate for residency, another study concluded that the performance during the residency interview is the most important factor (Pick et al., 2013).

Understanding the factors used by residency programs to rank candidates could make a great deal of difference. Knowing these factors could allow candidates the opportunity to plan for residency beforehand; this helps ensure they excel during the residency application process. In this study, we hypothesize that identifying the characteristics of a good pharmacy residency candidate helps applicants prepare for the residency application process and increase their chance of matching with a hospital. The study identifies the characteristics that residency program directors and preceptors use to define an ideal residency candidate.

## 2 Methods

This is a cross-sectional, descriptive study that surveyed the directors and preceptors of SCFHS accredited pharmacy residency programs across the Kingdom of Saudi Arabia. The study surveyed all current and previous residency program directors and preceptors of SCFHS accredited residency programs in Saudi Arabia. An electronic questionnaire was adapted by the study’s authors and reviewed by four experienced faculty in post-graduate pharmacy training. Some of the questions were taken from a study conducted by Hillebrand and colleagues (Hillebrand et al., 2015). Each faculty member independently revised the content of the questionnaire for clarity, consistency, and relevance to the study objectives.

The questionnaire consists of two sections: (A) demographic characteristics and (B) characteristics of the residency candidates. Section (A) is comprised of 12 questions assessing the directors and preceptors’ demographic information. This information includes questions related to their residency programs. Section (B) is comprised of 22 questions that evaluate applicant characteristics that are considered by the participants during the candidate selection process. The answers in section (B) are based on the five-point Likert scale [strongly agree (1), agree neutral (2), neutral (3), disagree (4), strongly disagree (5)].

All residency program directors and preceptors who participated in the selection process in Saudi Arabia were invited to respond to this self-administered questionnaire. To move forward in the survey, the participants were required to respond to each question; a respondent could stop the survey at any time. The survey was created using Google Forms, online survey software, and sent to the participants electronically over a five-month period (May 1, 2020 – September 30, 2020). If they did not complete the entire questionnaire, respondents were excluded.

A descriptive analysis was performed to assess the frequency and percentage of respondents’ demographic characteristics and responses to each criterion. The responses on the 5-point Likert scale were visualized as a Gannt chart using Tableau Software. For each question, these responses were reported as the mean and percentage of agreement or disagreement. Data management and statistical analyses were carried out using SAS version 9.4 statistical software (SAS Inc., Cary, North Carolina).

## 3 Results

After five months of data collection (May 1, 2020 – September 30, 2020), 78 of the directors and preceptors responded to the survey. The 68 responses that were completely filled out by current directors and preceptors were included. The study participants represent the geographic diversity of residency program directors located in Saudi Arabia. All of the current program directors and preceptors from SCFHS accredited residency programs across Saudi Arabia were contacted via email and sent an introductory email and electronic survey. Overall, 36 of the directors responded to the survey which represents (65%) of the total program directors in Saudi Arabia. That is, there are approximately 55 PGY-1 and PGY-2 residency program directors in Saudi Arabia. The remaining respondents were the preceptors from SCFHS accredited residency programs across Saudi Arabia. Overall, most of the respondents (60%) were located in the central region followed by the western region (32%). The vast majority of the respondents were directly involved in the resident selection process (89%). Respondents varied in their residency program types and levels as they were engaged in post graduate year 1 (PGY-1) (39%), in PGY-2 (11%), and in PGY-1 and PGY-2 programs (48%) (Table 1).

**Table 1:** Demographic Characteristics of the Respondents and Affiliated Residency Programs.

**Variable**	**No. (%)**
Sex	
Male	34 (50.0)
Female	34 (50.0)
Role in residency program	
Program director	36 (52.9)
Clinical preceptor	32 (47.1)
Years of experience	
≤ 1 year	13 (19.1)
2 years	12 (17.6)
3 years	15 (22.1)
4 years	8 (11.8)
≥ 5 years	20 (29.4)
Director/preceptor received pharmacy residency certificate	
Yes	55 (80.9)
No	13 (19.1)
Director/preceptor is involved in the selection process	
Yes	61 (89.7)
No	7 (10.3)
Type of residency program	
PGY1 (R1 and R2)	27 (39.7)
PGY2 (R3)	8 (11.8)
PGY1 and PGY2 (R1, R2, and R3)	33 (48.5)
Accreditation status of the residency program	
SCFHS and ASHP	25 (36.8)
SCFHS only	43 (63.2)
Geographic region of the residency program	
Central	41 (60.3)
Western	22 (32.3)
Eastern	2 (2.90)
Northern	0 (0.00)
Southern	3 (4.40)
Years since the establishment of the residency program	
≤ 1 year	12 (17.7)
2–3 years	14 (20.6)
4–5 years	9 (13.2)
>5 years	33 (48.5)
Number of positions offered by the residency program	
1 position	19 (27.9)
2 positions	9 (13.2)
3 positions	2 (3.00)
4–5 positions	18 (26.5)
≥ 6 positions	20 (29.4)
Residency program is affiliated with a pharmacy school	
Yes	22 (32.4)
No	46 (67.6)
**Total**	**68 (100)**

Overall, the survey assessed several factors that are prioritized during the resident’s selection process. Using the mean response score from the results of the Likert scale [strongly agree (1), agree neutral (2), neutral (3), disagree (4), strongly disagree (5)] — indicated that candidate’s performance during the interview (1.5), their professional appearance (1.5), an alignment between a candidate’s interests and the program focus (1.6), and previous hospital experience (1.8) mattered most. According to the respondents, these criteria were the most valued candidate attributes. In addition, language-related factors were highly valued in the selection process. In particular, English language and writing skill proficiencies were determined based on the quality of residency application documents.

**Fig. 1:**
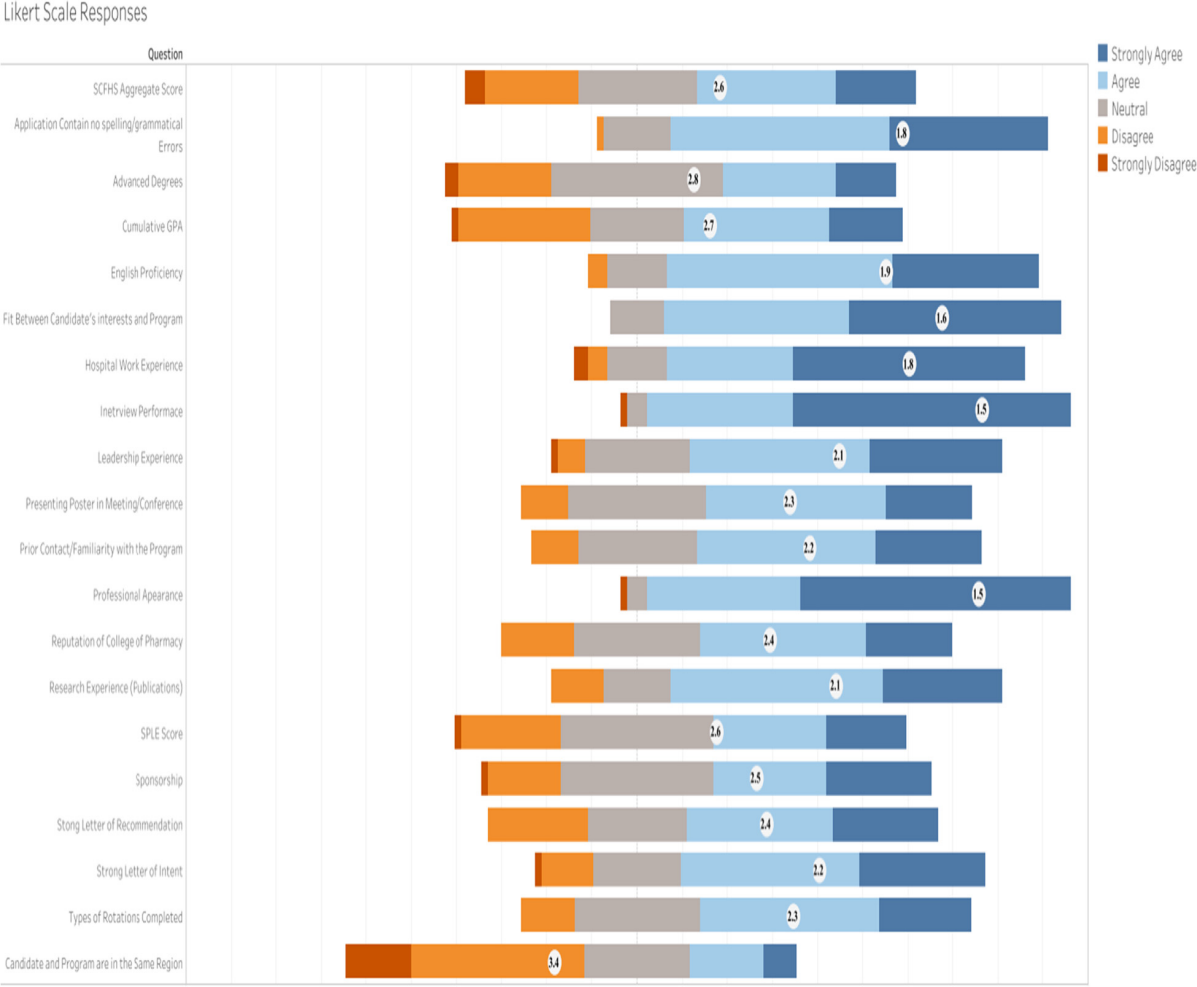
Factors Used in Evaluating Residency Applicants. Although the Likert Scale ranks responses from strongly agree to strongly disagree [strongly agree (1), agree neutral (2), neutral (3), disagree (4), strongly disagree (5)], this figure reverses these categories. The actual respondent scores are unaffected, but they are presented here in the opposite order when compared to the survey.

**Fig. 2:**
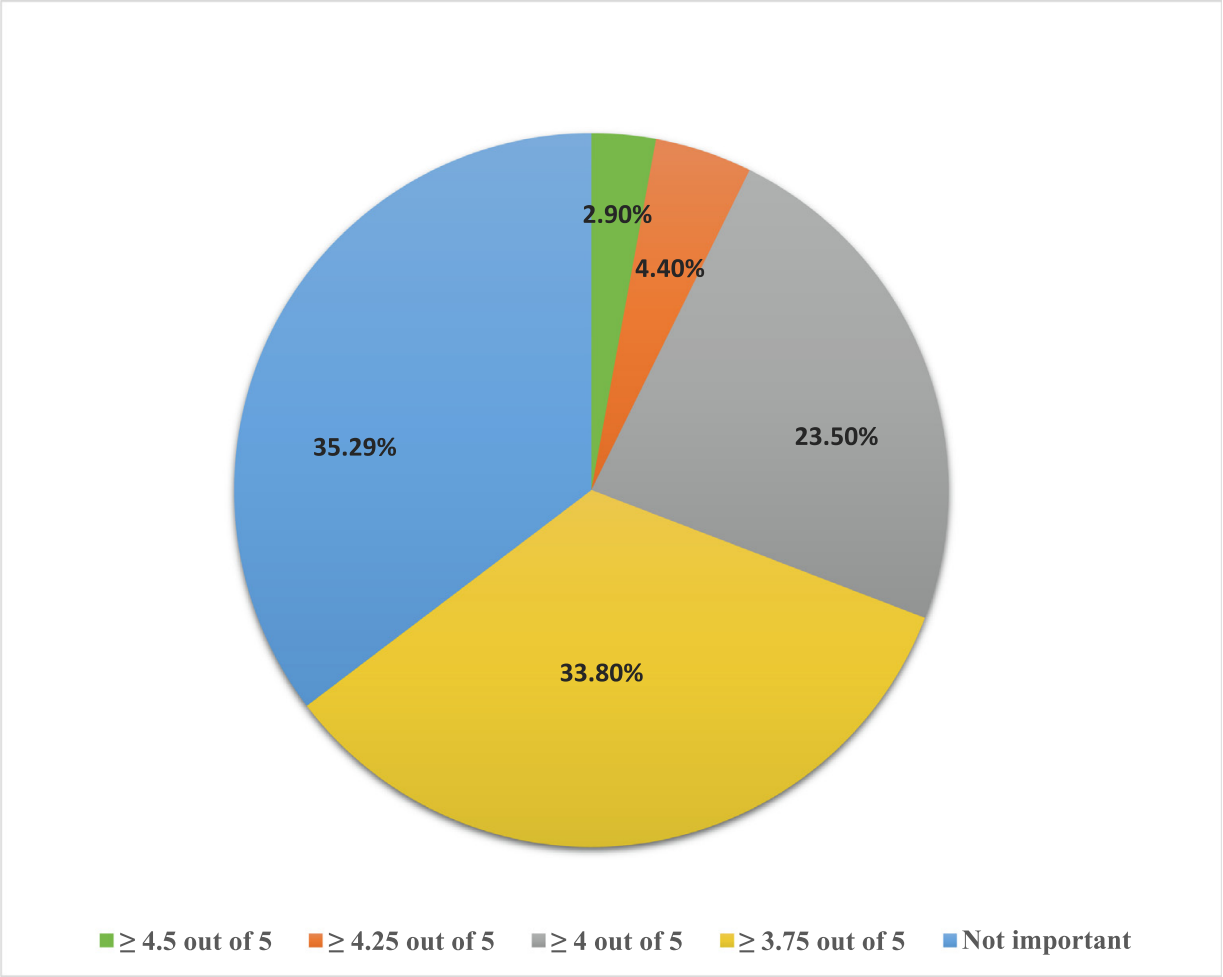
Minimum GPA Required Prior to Residency Application.

More than 52% of the respondents did not consider the region a candidate came from as an important factor in the selection process. The majority of the respondents were either neutral or disagreed with the idea that candidates should possess advanced degrees. Moreover, of all of the selection criteria, a candidate’s cumulative pharmacy school GPA was ranked at the bottom of the hierarchy (Fig. 1). Although the SPLE exam is a cornerstone for pharmacists obtaining a license, the SPLE and SCHFS scores did not play an important role (2.8) in the selection process — especially when compared to other factors. The mean scores of the other factors evaluated are displayed in the Gannt chart (Fig. 1). Slightly less than two thirds (65%) of respondents considered GPA prior to the residency application important (Fig. 2). 34% of respondents considered 3.75/5 or greater to be the minimum acceptable GPA for those applying to residency programs; 24% considered 4/5 or greater as the minimum (Fig. 2).

**Fig. 3:**
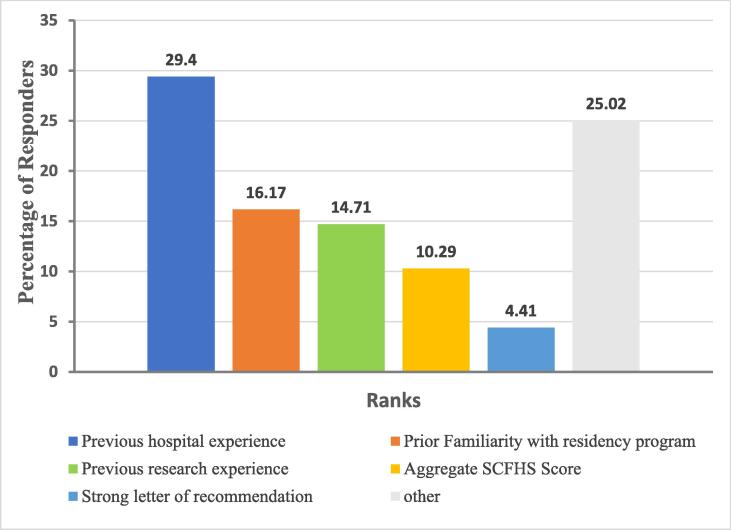
Top Five Factors Considered in the Selection of Residency Applicants. Other includes: SPLE score, GPA, letter of intent, types of rotations completed, previous leadership experience, poster presentations during scientific meetings, completion of another advanced degree, well written application, performance during the interview, professional appearance during the interview, English language proficiency, sponsorship status, reputation of the candidate’s college of pharmacy, whether the candidate’s school and residency program are located in the same region, and alignment between candidate’s interests and residency program.

When the directors and preceptors were asked to rank the factors based on their importance in the candidate selection process, previous hospital experience ranked first (29%) followed by a pre-existing relationship with the residency program (16%). Research experience (14%), SCFHS aggregate score (10%), and letters of recommendation (4.4%) also ranked among the most important factors (Fig. 3).

## 4 Discussion

We aimed to identify the characteristics that residency program directors or preceptors use to identify an ideal pharmacy residency candidate. We believe that identifying these characteristics can help pharmacy residency applicants in Saudi Arabia prepare for the residency application process.

By querying pharmacy program directors, several studies conducted in the US investigate which factors matter the most in the selection of pharmacy residency (Gohlke et al., 2014, Pick et al., 2013). Pick et al (2013) conducted a survey to identify the factors considered by residency directors in pharmacy residency selection. In their rankings, the residency interview was most important (67%) followed by letters of recommendation (9%) and then letters of intent (4%) (Pick et al., 2013). Another study conducted by Gohlke et al. (2014) focused on the characteristics that make an ideal PGY-1 pharmacy residency candidate. When residency program directors were asked to rate the qualities, they desired in a PGY-1 candidate, familiarity with the school of pharmacy was valued most — followed by previous hospital experience. They also ranked modifiable characteristics, which they defined as “factors that the candidate has influence over once accepted into a college of pharmacy”. Excellent letters of recommendation and compelling letters of intent ranked highly followed by previous experience with the same institution (Gohlke et al., 2014). On the other hand, previous publications and poster presentations were the lowest ranked.

In this study, when selecting a candidate for a residency position, most of the preceptors and program directors claim that previous hospital experience matters. Indeed, previous hospital experience ranked first with 29% of respondents choosing it as the most important factor. Pick and colleagues, reported that previous work experience (the survey did not specify if this refers to hospital or more general experience) ranked third among the most important characteristic in the residency process (4% of the of respondents consider it as the most important factor) (Pick et al., 2013). Gohlke et al. (2014) found that prior hospital experience is an important quality (Gohlke et al., 2014). In general, previous hospital experience conveys a candidate’s strong interest in the profession and constitutes evidence of a foundation of knowledge that increases one’s chances of successfully completing a residency program. Obviously, this factor disadvantages recent graduates.

In this study, familiarity with the program ranked second and previous research experience came in third. Prior familiarity with the residency program ranking second emphasizes how important it is to carefully select rotations during the last year of pharmacy school. Also, students should be encouraged to volunteer in these institutions to increase their knowledge of that program, form meaningful relationships, and make a good impression. It also serves as an opportunity for the students to determine whether this environment is suitable. Therefore, the internship year may be considered as a great time for pharmacy interns to build up this experience. In addition, PGY-1 pharmacy residents may also build up this experience by taking a rotation in their desired PGY-2 residency program. After all, students have different goals and ambitions when pursuing residency positions. Gohlke et al. (2014) also found that prior experience with the applicant is an important modifiable factor. In our study, a candidate’s previous research experience is also highly valued. Conversely, Gohlke et al (2014), report that previous research experience and poster presentations mattered least. Based on our findings, we encourage Saudi students to establish productive relationships with their mentors and take advantage of research opportunities. Doing so helps a candidate distinguish themselves from other applicants. Moreover, schools, mentors, and faculty members must assist students interested in residency positions to get involved in clinical research that extends beyond graduation project courses. That is especially important because — of a portfolio’s 20 possible points — 4 points are awarded for research activities.

In addition, residency programs seem to value letters of recommendation highly (Gohlke et al., 2014, Pick et al., 2013). This reinforces, then, the importance of providing preceptors, mentors, and faculty members with the resources they need to write a proper letter of recommendation. In our study, respondents reported that having a strong letter of recommendation is an important factor. Other, earlier studies concluded that letters of recommendation from preceptors and clinical faculties are preferred (Pick et al., 2013). Still, in our study, we did not specify the source of the letters of recommendation.

The respondents, when asked about “performance during the interview” — they said it was important, but it was not ranked among the top five factors. Indeed, we expected that the residency interview to be one of the top five factors — given that applicants are already pre-screened before they reach the residency interview stage. Several schools in the US recognize how critical it is to prepare for the residency interview. Therefore, they offer “mock interviews” and other preparatory resources for students who are interested in enrolling in residency programs (Buckley et al., 2018, Caballero et al., 2012, Koenigsfeld et al., 2012). By posing the most frequently asked questions during “mock interviews”, these resources familiarize students with the residency interview process. To our knowledge, mock interviews and other preparatory resources are not regularly offered in Saudi pharmacy schools. We strongly encourage pharmacy schools to provide such resources — even if the number of students interested in residency might comprise a small percentage of the student body.

In Saudi Arabia, pharmacy schools focus more on preparing the students for the SPLE exam, which is required for obtaining a pharmacist license. This is understandable given that the pass rate on the exam affects a school’s reputation but also because the SPLE score comprises 50% of the SCFHS aggregate score. Of course, this aggregate score ultimately decides who is promoted to the residency interview stage. In our study, according to the residency program directors and preceptors surveyed, the Likert scale did not show that the SPLE score was an important factor. Moreover, the SPLE score did not rank among the top five factors. Still, the SCFHS aggregate score was the fourth most important factor (10.29%), which is heavily affected by the SPLE score. For that reason, schools should offer mock SPLE exams. Perhaps, then, more attention could also be given to those interested in residency — since the higher the score they receive, the higher their SCFHS aggregate score.

One of the main strengths of our study was the inclusion of the majority of the program directors (around two-thirds of the total program directors). However, the response rate among the preceptors cannot be identified given the nature of electronic surveys. Another strength is the inclusion of preceptors who were heavily involved in the selection process of residency candidates, which was not the case with previously published studies. This study is not without limitations. First, it is possible that we missed some characteristics that the residency programs might also consider important. Second, we did not specify in our survey the type of rotations a student performed. It is conceivable, then, that some rotations are preferred by some residency programs or for certain residency positions. Finally, we did not specify the source of the letters of recommendation. It is possible that a letter of recommendation from clinical preceptors are preferred by some programs.

## 5 Conclusion

The existing literature lacks studies that identify ideal candidates’ characteristics for residency programs in Saudi Arabia. Our study highlights the aforementioned characteristics by surveying program RPDs and preceptors. Our goal is to help ensure candidates are well-prepared for the application process. Using the Likert Scale, we found that candidates’ performance during the interview, their professional appearance, alignment between a candidate’s interests and the program’s focus, and previous hospital experience are the most valued candidate attributes. We report that previous hospital experience, familiarity with the program, research experience, SCFHS aggregate score, and letters of recommendation are considered the top five factors.

To increase their chances of success, residency candidates should be encouraged to train in clinical or hospital settings as well as to plan ahead so they can receive the training in hospitals that host the programs they are most interested in. Students must contemplate and then decide whether a program aligns with their specific career goals. In addition, pharmacy interns and students should work on developing research skills and try to conduct research during internships or pharmacy school. Again, pharmacy colleges should provide mock interviews to students who are interested in applying to residency programs. The fact remains that there is still a need for studies that compare the perceptions of pharmacy interns with those of residency program directors and preceptors.

## Funding

This research did not receive any specific grant from funding agencies in the public, commercial, or not-for-profit sectors.

## Declaration of Competing Interest

The authors declare the following financial interests/personal relationships which may be considered as potential competing interests: Dr. Alhossan and Dr. Aseeri are affiliated with residency programs that received our survey, but they did not participate in completing the questionnaire. The other authors have nothing to disclose.
